# Evaluation of the Antimicrobial Efficacy of Cerium Oxide Nanoparticles and Chlorhexidine Gluconate Against Enterococcus faecalis in Root Canal Systems: Protocol for an In Vitro Study

**DOI:** 10.2196/77998

**Published:** 2026-03-02

**Authors:** Bhuvaneshwari Shrikant Khanadali, Anuja Ikhar

**Affiliations:** 1 Department of Conservative Dentistry and Endodontics Sharad Pawar Dental College Datta Meghe Institute of Medical Sciences Sawangi (Meghe), Maharashtra India

**Keywords:** nanoparticles, intracanal medicament, chlorhexidine gluconate, Enterococcus faecalis, biofilm, cerium oxide nanoparticles, single-rooted premolar

## Abstract

**Background:**

*Enterococcus faecalis* is a facultative anaerobic bacterium frequently associated with persistent root canal infections and endodontic treatment failures. Its resistance is primarily attributed to its ability to form robust biofilms, survive under harsh conditions, and penetrate deep into dentinal tubules. Chlorhexidine gluconate (CHX), commonly used as an intracanal medicament, exhibits broad-spectrum antimicrobial activity; however, its limited ability to eliminate biofilms, potential cytotoxicity, and restricted dentinal penetration pose significant drawbacks. In recent years, nanotechnology has introduced promising alternatives such as cerium oxide nanoparticles (CeO_2_-NPs), which exhibit unique redox properties, reactive oxygen species scavenging, and antimicrobial action due to their nanoscale size and surface chemistry.

**Objective:**

This study aims to evaluate and compare the antimicrobial efficacy of 0.05% CeO_2_-NPs, 2% CHX, and their combination against *E. faecalis* in root canal systems.

**Methods:**

Forty-eight extracted human single-rooted premolars will be decoronated, instrumented, and sterilized. The root canals will be inoculated with *E. faecalis* and incubated for 21 days to allow biofilm formation. The specimens will then be divided into four groups (n=12 per group), each receiving one of the following treatments: (1) 0.05% CeO_2_-NPs, (2) 2% CHX, (3) a combination of CeO_2_-NPs and CHX, and (4) sterile water as the negative control. After the medicaments are applied for 5 days, microbial sampling will be conducted, and the remaining viable bacteria will be quantified using colony-forming unit (CFU) counts on selective agar.

**Results:**

Ethics approval was obtained from the institutional ethics committee of Datta Meghe Institute of Higher Education and Research, Wardha (approval DMIHER(DU)/IEC/2025/546) in January 2025. As of January 2026, laboratory procedures, including specimen preparation, microbial inoculation, and data collection, have not yet commenced. Data collection is planned to begin in February 2026 and is expected to be completed by April 2026. Data analysis will follow immediately thereafter, with results anticipated to be submitted for publication in mid-2026. As this is an in vitro study, no participant recruitment, funding allocation, or clinical trial registration is applicable. It is expected that the combination of CeO_2_-NPs and CHX will result in the greatest reduction in bacterial load, followed by CeO_2_-NPs alone, CHX alone, and sterile water. These expected outcomes will be assessed based on comparative CFU counts for each treatment group.

**Conclusions:**

This study aims to provide insight into the antimicrobial potential of CeO_2_-NPs either alone or in combination with CHX against *E. faecalis*. If proven effective, CeO_2_-NPs could represent a novel and biocompatible adjunct or alternative to conventional intracanal medicaments, contributing to improved disinfection protocols and better clinical outcomes in endodontic therapy.

**International Registered Report Identifier (IRRID):**

PRR1-10.2196/77998

## Introduction

### Background

Root canal treatment success depends on the complete elimination of microorganisms from the root canal system and the prevention of reinfection. Despite advancements in endodontic techniques, microorganisms such as *Enterococcus faecalis* frequently persist in the root canal, contributing to treatment failure [1]. *E. faecalis* is particularly resilient due to its ability to survive in nutrient-depleted environments, penetrate deeply into dentinal tubules, and form biofilms [2]. These biofilms protect the bacteria from antimicrobials, making eradication extremely challenging [3].

Chlorhexidine gluconate (CHX) has been a gold standard intracanal medicament in endodontics, valued for its broad-spectrum antimicrobial properties and ability to bind to dentin, providing long-lasting antibacterial effects [4]. However, CHX is not without limitations. Its cytotoxicity, potential to cause discoloration, and inability to penetrate deep into dentinal tubules have driven researchers to explore alternative options [5]. Cerium oxide nanoparticles (CeO_2_-NPs) offer unique characteristics that distinguish them from conventional antimicrobial agents. These nanoparticles have dual oxidation states (Ce^3+^ and Ce^4+^) that enable them to generate reactive oxygen species, disrupt bacterial biofilms, and exhibit potent antimicrobial activity [6]. Furthermore, their nanoscale size allows for enhanced penetration into dentinal tubules, which is crucial for addressing *E. faecalis* located in deeper layers of the tooth substrate [7]. Additionally, CeO_2_-NPs demonstrate excellent biocompatibility, making them a promising candidate for clinical use [8].

This study addresses the persistent challenge of eliminating *E. faecalis*, a key contributor to root canal failure due to its resistance, dentinal invasion, and survival capabilities. Although CHX is a widely used intracanal medicament, its limitations, including cytotoxicity, discoloration, and limited penetration into dentinal tubules, necessitate alternative solutions [9].

CeO_2_-NPs offer promising advantages, including reactive oxygen species generation, biofilm disruption, nanoscale penetration, and superior biocompatibility. A comparative evaluation of CeO_2_-NPs and CHX is essential to determine their relative efficacy in targeting *E. faecalis* and improving clinical outcomes in endodontic treatments. Therefore, the aim of this study is to evaluate and compare the antimicrobial efficacy of 0.05% CeO_2_-NPs, 2% CHX, and their combination against *E. faecalis* in an in vitro root canal infection model (extracted human teeth).

### Objectives

The objectives of our study are as follows: (1) to assess the antimicrobial efficacy of 2% CHX as an intracanal medicament by measuring its ability to reduce the colony-forming units (CFUs) of *E. faecalis* in dentinal tubules, (2) to assess the antimicrobial efficacy of 0.05% CeO_2_-NPs as an intracanal medicament by measuring its ability to reduce CFUs of *E. faecalis* in dentinal tubules, and (3) to assess the antimicrobial efficacy of 0.05% CeO_2_-NPs and 2% CHX as an intracanal medicament by measuring its ability to reduce CFUs of *E. faecalis* in dentinal tubules.

## Methods

### Study Design and Settings

This study adopts an in vitro design to simulate and control the root canal environment for standardized and reproducible evaluation of antimicrobial efficacy. This design is appropriate, as it eliminates confounding variables present in clinical conditions and enables precise comparison among test groups. The selection of this design is based on feasibility, ethical considerations (no human/animal subjects involved), and the objective to isolate the effects of the medicaments on *E. faecalis*. This study was conducted in the Department of Conservative Dentistry and Endodontics, Sharad Pawar Dental College & Hospital, Sawangi (Meghe), Wardha.

### Ethical Considerations

Ethical clearance for this in vitro study was obtained from the institutional ethics committee of Datta Meghe Institute of Higher Education and Research, Wardha, India (approval DMIHER(DU)/IEC/2025/546). All extracted teeth will be collected from the department of oral surgery, ensuring that they are not at risk of harm due to the use of teeth in research. This study will follow strict guidelines for ethical handling of human tissues. As this is an in vitro study, no participant recruitment, funding allocation, or clinical trial registration is applicable. Data will be stored on a password-protected computer accessible only to the principal investigator and supervisor. Data privacy and confidentiality will be maintained in accordance with institutional research policies.

### Study Items

In this study, we use 48 freshly extracted, single-rooted human premolar teeth extracted for orthodontic or periodontal reasons. Single-rooted premolars provide a uniform root canal system ideal for standardizing canal instrumentation and medicament application. Extracted human teeth offer realistic dentinal tubule structures necessary for valid medicament assessment.

### Inclusion Criteria

The inclusion criteria are as follows: (1) single-rooted human premolars with fully developed apices; (2) teeth with a single canal confirmed radiographically; (3) teeth free from caries, restorations, cracks, fractures, or resorption; and (4) no prior endodontic treatment.

### Exclusion Criteria

The exclusion criteria are as follows: (1) multi-rooted teeth or teeth with complex canal morphology, (2) teeth with calcified canals or accessory canals, and (3) teeth with structural defects (eg, resorption, fractures).

### Sample Size

The sample size is calculated based on a previous study by Pandey et al [10] by using mean CFU values and SDs, as shown in Textbox 1.

**Sample size calculation.**

**Mean colony forming unit (CFU) values and SDs**

Mean CFU in control = 40.545

Mean CFU in test group = 28.091

Standard deviation (σ1) = 11.021

Standard deviation (σ2) = 10.270

Desired difference (∆) = 12.454

Power=80%

α=.05

**Sample size calculation formula**

N = (Z_1−α/2_ + Z_1−β_)^2^(σ_1_^2^ + σ_2_^2^) / (μ_1_−μ_2_)^2^

The notation for the formulae are as follows:

- n_1_ = sample size of group 1
- n_2_ = sample size of group 2
- σ_1_ = standard deviation of group 1
- σ_2_ = standard deviation of group 2
- Δ = difference in group means
- κ = ratio = n_2_ / n_1_
- Z_1_-α/_2_= 2-sided Z value (eg, Z = 1.96 for 95% CI)
- Z_1_-β= power

For detecting the mean difference of 12.454, that is, ∆ = 40.545-28.091 = 12.454

K = 1

N = (11.021*11.021 + 28.091*28.091)(1.96+0.84)^2^ / 12.454*12.454

= 11.47 = 12 samples needed in each group (12*4=48)

**Result:** 12 samples per group × 4 groups = 48 total samples

**Level of significance:** 5% ( 95% CI)

### Sampling Method and Group Allocation

Simple random sampling was performed from the pool of the eligible extracted teeth. The intervention for each group is allocated as shown in Table 1.

**Table 1:** Intervention for each group of eligible extracted teeth.

Group	Intervention	Samples, n
Group 1	2% chlorhexidine gluconate (CHX)	12
Group 2	0.05% cerium oxide nanoparticles (CeO_2_-NPs)	12
Group 3	0.05% CeO_2_-NPs + 2% CHX	12
Group 4	Sterile water (control)	12

### Medicaments

The following medications are used.

1. CHX (2%)
2. CeO_2_-NPs (0.05%): <25 nm particle size, 0.5 mg/mL dispersion in sterile distilled water, prepared using ultrasonication for 1 minute for uniform dispersion, and then a thickening agent methyl cellulose is added.
3. Combination group: equal volume of 0.5 mg/mL CeO_2_-NPs and 2% CHX is mixed and ultrasonicated for 1 minute for uniform dispersion, and then a thickening agent methyl cellulose is added.

### Tooth Preparation

The eligible extracted teeth are prepared as follows.

1. Decoronation: standardized to 6-mm root segments using a diamond disc (Mani Inc).
2. Canal preparation: Gates-Glidden drills (#3 and #4) for standardization of canal diameter.
3. Smear layer removal: 2 mL of 17% ethylenediaminetetraacetic acid (5 min) followed by 5 mL of 3% sodium hypochlorite (5 min) and then rinsed with 5 mL of sterile distilled water.
4. Sterilization: double autoclave cycle, including immersion in Mueller-Hinton broth for the second cycle.

### Root Canal Infection

The root canals will be inoculated with *E. faecalis* and incubated for 21 days to allow biofilm formation. To induce root canal infection, the following agents are used: (1) *E. faecalis* (ATCC 29212) cultured in Mueller-Hinton broth and (2) dentin blocks incubated with 1 mL of 0.5 McFarland bacterial suspension for 21 days at 37 °C, with fresh inoculum every 48 hours.

### Application of Medicaments

Medications are applied by introducing 100 µL of the medicine into the samples by using a 27-gauge syringe, and then the samples are sealed with sticky wax to simulate clinical conditions. Thereafter, the samples are incubated under anaerobic conditions at 37 °C for 5 days.

### Posttreatment Sampling

Ater 5 days of incubation, dentin shavings will be collected at 400 µm depth by using Gates-Glidden drill size #5. The shavings will be suspended in Mueller-Hinton broth and serially diluted. Approximately 50 µL will be plated on Mueller-Hinton agar and incubated for 24 hours at 37 °C.

### Colony Counting

CFUs are counted manually or using a digital colony counter (Remi Lab Instruments).

### Outcome Measures

The primary outcome is quantitative comparison of the antimicrobial efficacy measured by CFU counts. The secondary outcome is the comparative effectiveness of the combination group vs individual agents and sterile water.

### Data Management

All data collected during the study will be manually recorded in prestructured data collection sheets and later transferred into a secure digital database. Double data entry will be employed to minimize errors. Data will be stored on a password-protected computer accessible only to the principal investigator and supervisor. Data privacy and confidentiality will be maintained in accordance with institutional research policies.

### Descriptive Statistics

The mean, standard deviation, and range will be calculated for CFU counts in each group. The graphical representation (bar graphs or box plots) will be used to visualize the microbial reduction across groups.

### Inferential Statistics

To evaluate the null hypothesis of no significant difference among groups, inferential statistical analysis will be performed. The Shapiro-Wilk test will be used to assess the normality of the CFU data. If the data are nonnormally distributed, appropriate transformations or nonparametric alternatives will be applied. One-way ANOVA will be employed to compare mean CFU counts across the 4 groups, followed by Tukey Honest Significant Difference test for pairwise comparisons. Levene test will be used to assess the homogeneity of the variances, and if ANOVA assumptions are violated, the Kruskal-Wallis test will be considered. A *P* value of less than .05 will be considered statistically significant. All analyses will be conducted using SPSS Statistics for Windows (version 27.0; IBM Corp) and GraphPad Prism (version 7.0), which will also be used for data visualization and assumption verification.

## Results

This study is in the protocol development phase, and no experimental results are available at this time. Following completion of the laboratory phase, the primary outcomes will include quantitative assessment of bacterial reduction based on CFU counts for each experimental group. Ethical approval was obtained from the institutional ethics committee of Datta Meghe Institute of Higher Education and Research, Wardha (approval DMIHER(DU)/IEC/2025/546) in January 2025. As of January 2026, laboratory procedures, including specimen preparation, microbial inoculation, and data collection, have not yet commenced. Data collection is planned to begin in February 2026 and is expected to be completed by April 2026. Data analysis will follow immediately thereafter, with results anticipated to be submitted for publication in mid-2026. The results will be analyzed to compare the antimicrobial efficacy of 2% CHX, CeO_2_-NPs, their combination, and sterile water (control). Outcome data will be presented in both tabular and graphical formats, including groupwise mean CFU counts, SDs, and *P* values derived from intergroup comparisons.

## Discussion

Persistent root canal infections, particularly those involving *E. faecalis,* remain a significant challenge in endodontic therapy. This is primarily attributed to the microbe’s deep dentinal penetration and resistance to standard antimicrobial agents. Despite advancements in mechanical instrumentation and irrigation protocols, complete disinfection of the root canal system is difficult to achieve, necessitating the exploration of more effective intracanal medicaments. A 21-day incubation period was selected to allow sufficient time for *E. faecalis* to colonize and penetrate the dentinal tubules, achieving a mature and resilient intraradicular infection model. Previous studies have demonstrated that *E. faecalis* requires extended incubation periods—typically ranging from 14 to 21 days—to reach substantial colonization and simulate persistent root canal infections [1,11]. Sanju et al [11] specifically used a 21-day incubation model to assess the antimicrobial effects of intracanal medicaments at deeper dentin levels, affirming that this duration provides consistent and clinically relevant bacterial invasion. This time frame enables the formation of bacterial populations that are more difficult to eradicate, thus providing a rigorous test for evaluating antimicrobial interventions.

Several recent studies have emphasized the potential of CeO_2_-NPs as a promising antimicrobial agent. CeO_2_-NPs have demonstrated significantly higher efficacy than calcium hydroxide, achieving a 66.9% reduction in *E. faecalis* CFUs at 400-µm dentin depth [11]. This is particularly relevant in endodontics, where bacteria often penetrate deeply into dentinal tubules. The enhanced antimicrobial action of CeO_2_-NPs is attributed to their nanoscale size and surface reactivity, which allow for better penetration and biofilm disruption [11]. This study builds upon this foundational work by directly comparing the antimicrobial efficacy of 0.05% CeO_2_-NPs, 2% CHX, their combination, and sterile water within an in vitro root canal biofilm model. The deep dentin effectiveness of 2% CHX gel has been previously confirmed, underscoring its value as a standard agent in endodontic disinfection [12]. However, [12] also highlighted its limitations, particularly in eliminating biofilm-residing bacteria. CeO_2_-NPs have demonstrated broad-spectrum antimicrobial potential, attributed to their ability to generate reactive oxygen species and induce oxidative stress in microbial cells [13]. Their biofilm-disruption capacity has also been reported to be higher than that of CHX, prompting recommendations for further investigation into their combined use [14].

The potential value of combining CHX and CeO_2_-NPs lies in their distinct yet potentially complementary antimicrobial mechanisms. CHX exerts its antimicrobial effect by disrupting bacterial cytoplasmic membranes, leading to leakage of intracellular components and cell death while also binding to dentin to provide prolonged antibacterial activity [4,5]. CeO_2_-NPs, in contrast, operate via redox cycling between Ce^3+^ and Ce^4+^ oxidation states, facilitating the generation of reactive oxygen species and inducing oxidative stress within microbial cells [6,13]. Moreover, the nanoscale size of CeO_2_-NPs enables deeper penetration into dentinal tubules, enhancing access to areas where conventional agents may be less effective [7,8]. When used in combination, these agents may exert additive or synergistic effects, CHX compromising membrane integrity and CeO_2_-NPs amplifying oxidative damage thereby offering a multi-targeted antimicrobial approach. Clinically, such a combination could significantly improve root canal disinfection, especially against persistent organisms such as *E. faecalis* that inhabit deeper layers of dentin [1,2]. This approach may overcome the limitations of monotherapies and enhance long-term treatment outcomes. Consequently, evaluating this combination is both scientifically and clinically justified.

The anticipated outcomes of this study are expected to support and build upon previous findings in the field. By evaluating both individual and combined effects of CeO_2_-NPs and CHX, this study explores possible synergistic interactions, a topic previously suggested but not thoroughly investigated in the context of dentin-embedded *E. faecalis* [14].

A key strength of this study is the use of extracted human teeth, offering a realistic anatomical model that closely resembles clinical conditions. This enhances the translational value of the findings. The controlled in vitro design minimizes external variables and allows precise comparison between the treatment groups. Furthermore, focusing on *E. faecalis*, a pathogen well-documented in endodontic failures, increases the clinical relevance of the study outcomes [12]. The investigation of 0.05% CeO_2_-NPs as a novel nanotherapeutic agent adds innovation to the study, offering insights into future directions for endodontic disinfection strategies [11,13]. It is hypothesized that the CHX and CeO_2_-NP combination could improve antimicrobial efficacy, justifying further experimental exploration [14]. Although some constraints are inherent to in vitro designs, such models provide a controlled environment ideal for evaluating antimicrobial efficacy in a reproducible and standardized manner. This foundational research supports the potential of CeO_2_-NPs—alone or in combination with CHX—as effective agents against resistant endodontic pathogens and sets the stage for future in vivo investigations.

Although this study employs a controlled and standardized in vitro design to evaluate the antimicrobial efficacy of CeO_2_-NPs and CHX, it does not fully replicate the complex biological conditions present within the human oral cavity. The absence of host immune responses, salivary enzymes, and polymicrobial communities may influence bacterial behavior differently in vivo. Furthermore, factors such as biofilm heterogeneity, fluid dynamics, and interactions with periapical tissues are not represented in this model. Although the use of extracted human teeth enhances anatomical relevance, clinical extrapolation of the findings should be approached with caution. Future in vivo studies and randomized clinical trials are necessary to validate the translational potential of these agents under physiological conditions. This in vitro study aims to explore the antimicrobial potential of CeO_2_-NPs, alone and in combination with CHX, in the disinfection of root canal systems. By addressing the limitations of current medicaments and leveraging nanotechnology, this protocol may contribute valuable insights into future endodontic disinfection strategies. The findings, once available, will support the development of enhanced treatment protocols for persistent root canal infections caused by *E. faecalis*.

## Abbreviations

CeO_2_-NP: cerium oxide nanoparticle
CFU: colony-forming unit
CHX: chlorhexidine gluconate
